# Caregivers’ experiences, challenges, and needs in caring for people with dementia in India: a scoping review

**DOI:** 10.1186/s12913-024-12146-x

**Published:** 2024-12-30

**Authors:** Daniela Lillekroken, Liv Halvorsrud, Heidi Bjørge, Sailaxmi Gandhi, Palanimuthu T. Sivakumar, Alka Rani Goyal

**Affiliations:** 1 Department of Nursing and Health Promotion, Faculty of Health Sciences, Oslo Metropolitan University, PB 4 St. Olavs Plass, N – 0130, Oslo, Norway; 2 Department of Nursing, National Institute of Mental Health and Neurosciences, Bengaluru, Karnataka India; 3 Department of Psychiatry, National Institute of Mental Health and Neurosciences, Bengaluru, Karnataka India

**Keywords:** Dementia, Caregivers’ experiences, India, People with dementia, Scoping review

## Abstract

**Background:**

As the world’s most populous country, India faces a growing challenge in addressing dementia, in which advancing age remains the strongest risk factor. Approximately 8.8 million Indians over the age of 60 are currently affected by this condition. While much of the research on dementia caregiving focuses on Western societies, caregiving experiences are shaped profoundly by cultural and socioeconomic contexts, which vary significantly in India. Unique social norms, combined with limited resources, make the burden on dementia caregivers in India both distinct and understudied. This scoping review synthesized existing literature on the experiences, challenges, and needs of dementia caregivers in India, providing critical insights for developing culturally tailored support systems and informing public health initiatives.

**Methods:**

A comprehensive database search for literature was conducted in April 2022 and updated in September 2024 in six databases **(**Medline, Embase, PsycINFO, CINAHL, Web of Science and Epistemonikos). The databases were systematically searched for original qualitative and quantitative peer-reviewed studies conducted in India between 2000 and 2024. The scoping review was registered with the Open Science Framework and was reported in accordance with Preferred Reporting Items for Systematic Reviews and Meta-Analyses extension for Scoping Reviews Checklist (PRISMA-ScR).

**Results:**

After identifying, screening, and reviewing articles for eligibility, we critically appraised and included a sample of 27 studies in this review. The data synthesis process identified three main themes: (1) navigating the dual realities of caregiving: challenges and rewards for family caregivers in India, (2) gaps in support for home-based care: challenges faced by family caregivers in rural parts of India, and (3) addressing the present and future needs of caregivers for people with dementia in India.

**Conclusion:**

This scoping review reveals the experiences, challenges, and needs of caregivers for people with dementia, including caregiver burden, stress, and poor health among caregivers resulting from inadequate support, insufficient respite care, and a lack of information about dementia. Health and social services offering dementia care are critically needed in India, where population aging is imminent.

**Trial registration:**

https://doi.org/10.17605/OSF.IO/4MJDC.

**Supplementary Information:**

The online version contains supplementary material available at 10.1186/s12913-024-12146-x.

## Background

India, the world’s most populous country [1], is experiencing growth in its older adult population [2, 3]. The rapidly increasing number of older adults will also increase the need for healthcare and support for them, but the time to develop services to respond to the aging population and their needs remains limited [4].

The incidence of dementia increases exponentially with age, affecting over 55 million people worldwide, with nearly 10 million new cases registered each year [5]. In 2019, the global economic impact of dementia reached US $1.3 trillion. About half of this cost was attributed to care provided by informal caregivers, such as family members and close friends, who typically dedicate an average of five hours per day to caregiving and supervision [5].

Since the 1990s, studies have demonstrated that the prevalence of dementia in India has varied from 3.5% to 3.9%, with higher estimates reported in rural areas than in urban settings [6, 7]. Recently, Lee et al. [8] demonstrated that the estimated dementia prevalence for adults aged 60 and above in India is 7.4%, which means that 8.8 million Indians older than 60 years live with dementia, a figure expected to exceed 17 million by 2036 [8]. These recent figures were higher in rural than in urban contexts. Although these figures indicate that dementia is a significant cause of concern within the Indian context, there is generally a lack of awareness and understanding of dementia in most low- and middle-income countries, resulting in stigmatization and barriers to diagnosis and care that affect patients, caregivers, families, and societies, both psychosocially and economically [9]. Dementia’s socioeconomic impacts are indeed tremendous not only in low-and middle-income countries [10, 11], but also worldwide [12, 13]. To raise awareness about the condition and its consequences, in 2012, the World Health Organization (WHO) declared dementia a public health priority [14].

In India, although caregiving responsibilities and liabilities are primarily borne by individual families [15], awareness of dementia and its consequences for people with dementia (PwD) and their families remains poor [16]. Several government and private agencies have begun providing care to PwD in India, but the concept of dementia care outside the home remains relatively new [17]. Families, society, and other stakeholders tend to attribute dementia’s symptoms to ageing, and professional help for PwD and their family caregivers (FCGs) is seldom sought, as it is linked to stigma [16]. Because families assume caregiving roles and responsibilities to support PwD’s needs, the real care burden and societal cost of dementia in India remains obscured [18]. Poor or nonexistent healthcare and social services for PwD and their FCGs contribute to caregivers’ burden [7, 15]. In India, in particular, the care provided by FCGs to PwD is nearly 10 times greater than that provided by healthcare personnel, such as nurses, social workers, physicians, and psychologists [18]. The change in preference for nuclear families compared to the traditional joint family system and the rising number of working women in India, especially in urban communities [19], make it difficult to imagine how Indian society will provide sufficient care for PwD, primarily via FCGs, in the near future [20]. While healthcare personnel in India have a moderate level of knowledge about dementia, there is a need to further develop dementia care in order to meet the current and future demands of the country’s aging population [21, 22].

To the best of our knowledge, on the basis of a comprehensive search conducted on April 25, 2022, and updated on September 4, 2024, no review has specifically examined and synthetized the experiences of caregivers of PwD in India. This gap in the literature, along with the limited number of studies addressing care-related needs from the perspectives of FCGs and healthcare professionals, underscores a critical need for further research. Such research is essential if dementia care in India aims to efficiently support FCGs and healthcare personnel in providing dignified, person-centered care for PwD.

## Methods

### Research aim

This scoping review aims to examine the existing literature on the experiences of FCGs and healthcare personnel involved in the care of PwD in India.

### Study design

The scoping review was conducted following the PRISMA-ScR Checklist, developed by members of the Joanna Briggs Institute (JBI) and collaborating centers [23, 24]. The review was registered with the Open Science Framework (10.17605/OSF.IO/4MJDC). Scoping reviews capture a broad range of literature, including studies with different designs, relevant to addressing the topic of interest [25]. When conducting our review, we followed the five stages described by Arksey and O’Malley [25]: (1) formulation of the research question, (2) study identification, (3) study selection, (4) data charting, and (5) collating, summarizing, and reporting the results.

#### Stage 1: Formulation of the research question

The following research question guided the search for relevant literature to be included in the scoping review: What are the experiences, challenges, and needs of FCGs and healthcare professionals in caring for PwD in India?

#### Stage 2: Study identification

On April 25, 2022, with the guidance of a research librarian at Oslo Metropolitan University, we performed an advanced, systematic literature search in the CINAHL, Embase, Epistemonikos, Medline, PsycINFO, and Web of Science databases. The keywords developed by each database to index literature were searched for in the titles, abstracts, and main texts of studies by using the Boolean operator “AND”/”OR”. The keywords and phrases included in the literature search, were as follows: Dementia (dementia OR Alzheimer*) AND Caregivers or carer (family OR relative* OR spouse* OR families), (healthcare personnel OR nurse* OR doctor* OR physician* OR health care professional* OR healthcare worker*) AND Experiences (experience* OR perspective* OR living) AND India (India OR Indian population*). After the initial search on April 25, 2022, the first author set up email notifications from April 25, 2022, to March 30, 2023, to receive relevant and up-to-date information about new publications on the topic. The reference lists of the titles received were also screened for additional studies. Only two publications identified through these email notifications met the inclusion criteria [26, 27]. To ensure that the results were current, we conducted an additional search using the same keywords and phrases in the same databases on September 4, 2024. This search identified two more articles [16, 28] published between March 30, 2023, and September 4, 2024, that met the inclusion criteria and were thus included. An example of the updated search is presented in Supplementary File 1.

#### Stage 3: Study selection

Eligible publications were downloaded into EndNote version 21 [29] software and grouped by the first author. After the initial screening of the titles, duplicates were removed. The remaining publications were then screened to exclude editorials, abstracts, conference papers, posters, theses, commentaries, and secondary research and imported to Rayyan [30]. Publications were considered eligible based on the following inclusion criteria:

- Type of study: original qualitative or quantitative research studies
- Geographic location: studies conducted in India
- Participants: FCGs of PwD, and/or healthcare personnel involved in providing care to PwD
- Focus of study: studies that describe experiences related to providing home-based care or any other type of healthcare for PwD
- Language: studies published in English
- Publication date: studies published between January 2000 and September 2024

The publication period from January 1, 2000 to September 4, 2024, was chosen because healthcare professionals and researchers throughout India began networking about dementia in the late twentieth century paying close attention to dementia care and raising public awareness about dementia in India [31].

Publications were excluded if they focused on the experiences of FCGs or healthcare personnel caring for older adults with conditions other than dementia or if they were conducted in contexts outside of India. Additionally, studies exploring caregivers’ experiences of dementia care during the COVID-19 pandemic were excluded as the pandemic created unprecedented challenges and changes in healthcare delivery that were not reflective of standard dementia care. Including such studies could result in findings that are heavily influenced by the unique context of the pandemic, thus limiting the applicability and relevance of the results to nonpandemic situations. To ensure that the findings were generalizable and relevant to standard dementia care in India, we excluded these studies.

**Fig. 1:**
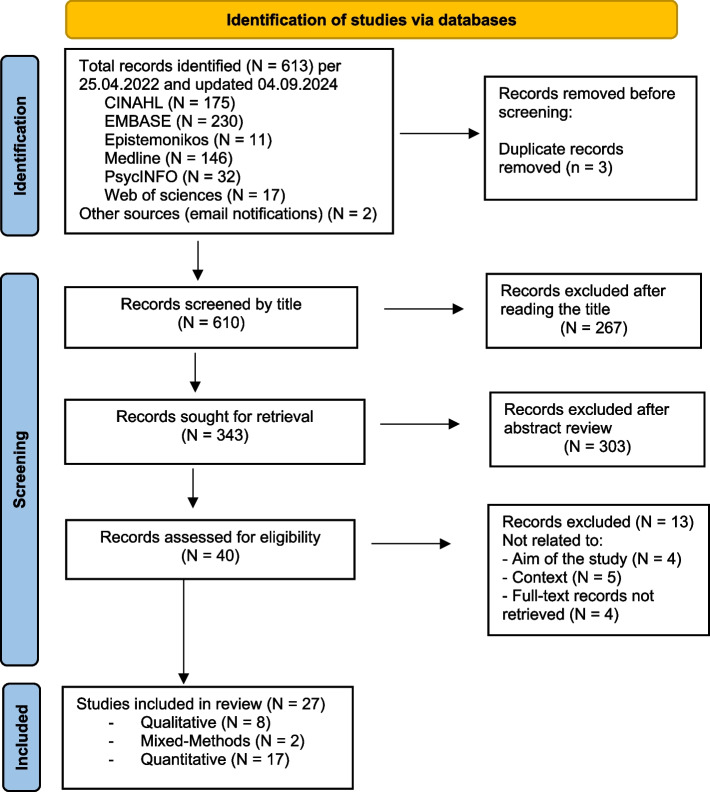
PRISMA flow diagram

Applying the inclusion and exclusion criteria, four authors (ARG, HB, LH, and DL) independently conducted an initial title and abstract screening to assess the relevance of the publications. The publications were then randomly and equally divided between two pairs of authors (ARG and DL; HB and LH) for full-text reading. Each author independently reviewed the publications assigned to their group and extracted key information from the relevant data. The authors met regularly to discuss their conclusions, ensuring a consistent approach to evidence extraction across all the studies included in the review. Disagreements were resolved through discussion and, if necessary, by consulting an author from the other pair of reviewers in order to reach a consensus. The process of study selection is presented in Fig. 1 [32].

#### Stage 4: Data charting

A data charting table was developed to record the following information about the studies: author(s)/year of publication, title, aim(s), study design, method(s), participants, setting, key findings regarding caregivers’ experiences, challenges, and needs in caring for PwD in India. Publications reporting the same study but describing different or new results were included as separate sources, such as the studies of Lamech et al. [33, 34] and Tomita et al. [35, 36].

The quality assessment of the publications was conducted by four authors in pairs (ARG and DL, LH and HB) using the Mixed Methods Appraisal Tool (MMAT) which facilitates the description and appraisal of five types of studies [37]. The MMAT includes a total of 25 criteria and two screening questions and appraises five categories of studies: qualitative, randomized controlled, nonrandomized, quantitative descriptive, and mixed methods. Each category has five core quality criteria rated as “Yes”, “No” or “Can’t tell”. The assessment of ethical standards was carried out following the guidelines proposed by Weingarten et al. [38] for evaluating ethics in systematic reviews. All studies met the quality-related criteria and were included for further assessment.

#### Stage 5: Collating, summarizing, and reporting results

All data from the included studies were extracted by two authors (ARG and DL) and cross-checked by two other authors (LH and HB). General information about these studies was obtained using a data extraction form, which is presented in Table 1. Data synthesis was performed following the JBI Manual [23]. The “Results” and “Findings” sections of the different studies were extracted and analyzed by the first author (DL) for qualitative studies and by the last author (ARG) for quantitative studies. The quantitative data were summarized and, together with the qualitative data, analyzed using thematic analysis inspired by Braun and Clarke [39]. Both the first and last authors independently read all articles and, by comparing the selected codes, identified relevant themes and categories. The analyses were then compared and discussed until a consensus was reached among the first, second, third, and last authors. The second and third authors (LH and HB) then cross-checked the analyses to ensure accuracy, and the analysis continued iteratively until all categories and themes were fully defined and described and until all six authors reached a consensus. Data analysis was performed manually.

**Table 1:** Characteristics of included studies in the review (*N* = 27)

Author(s)/Year/Ref. nr	Title	Study aim	Study design	Data collection methods	Sample, (size), mean age (years)	Study setting	Key findings
Anand et.al., 2016, [40]	Perceived caregiver stress in Alzheimer's disease and mild cognitive impairment: A case control study	To evaluate the perceived stress in caregivers of patients with AD and MCI compared to the perceived stress in caregivers of patients with other chronic illness	Quantitative RCT	Caregivers were interviewed using PSS. Patients were assessed using The Blessed Activity of Daily Living (ADL), MMSE and CDR. PSS were compared amongst both groups and correlated with severity of illness and ADL of the patients	Caregivers of patients with AD and MCI (*n* = 31), mean age (69). Caregivers of patients with chronical medical and psychiatric disorders (*n* = 30), mean age (56)	Memory clinic of Neurology Department of a tertiary hospital in Northern India/New Delhi (Urban area)	Caregivers for MCI patients had lower PSS scores than AD caregivers, but both scored significantly higher than caregivers for other chronic disorders. A significant correlation was found between perceived stress and ADL (*P* < 0.001), indicating that increased ADL dependency in AD/MCI patients was linked to higher PSS scores compared to other chronic disease caregivers
Baruah et al., 2020, [41]	Perspectives on Components of an Online Training and Support Program for Dementia Family Caregivers in India: A Focus Group Study	To identify the components and understand the acceptability of an online training and support program for dementia caregivers in India	Qualitative	Three focus group discussions. Two with caregivers of PwD and one with health professionals	Primary caregivers of PwD (*n* = 13), HCP (*n* = 10), mean age; caregivers (47), mean age HCP (34,9)	Caregivers attending a geriatric clinic at a tertiary hospital and a non-governmental organization involved in dementia care (ARDSI) in southern India/Bengaluru (Urban area)	Expectations for an online dementia management program varied, including information provision and caregiver support. HCP suggested using simple language, cultural relevance, and mobile-friendly interactive education. Challenges identified by FCGs and HCP included lack of time, difficulty in internet access, lack of digital awareness and difficulty in reaching rural populations
Basu et. al., 2022, [26]	Neuropsychiatric symptoms of dementia and caregivers’ burden: a study among Indian Caregivers	To evaluate relationship between neuropsychiatric symptoms of dementia and caregiver burden	Quantitative cross sectional	Dementia care-recipients’ NPS severity, and caregivers’ burden were assessed with NPI	Caregivers of PwD (*N* = 138), mean age (61)	A non-governmental organization providing dementia care in eastern India/Kolkata (Urban area)	Severity of NPS in PwD correlated with caregivers’ burden severity, with 66.6% of caregivers of patients with severe NPS experiencing a higher burden than those with less severe NPS
Danivas et al., 2016, [42]	An interpretative phenomenological analysis (IPA) of coercion towards community dwelling older adults with dementia: findings from Mysore studies of natal effects on ageing and health (MYNAH)	To find relevant themes of the lived experience of relatives as caregivers for PwD in view of their use of coercive measures in community setting in South India	Qualitative explorative	Individual interviews	Primary caregivers of PwD (*N* = 13), mean age (73)	Caregivers of community dwelling PwD who were identified in an epidemiological survey “Mysore study of Natal effects on Ageing and Health (MYNAH)” in Southern India/Mysore (Urban area)	Caregivers indicated physical and emotional burn-out, insufficient respite services, and community support. They experienced poor social and emotional health and limited personal activities and job opportunities Caregivers often used coercion methods like misuse of sedatives, seclusion, environmental restraint, and diet restriction. These methods were used to ensure PwD’s safety, management of behavioural issues and ADL
Dias et al., 2008, [43]	The Effectiveness of a Home Care Program for Supporting Caregivers of Persons with Dementia in Developing Countries: A Randomised Controlled Trial from Goa, India	To develop and evaluate the effectiveness of a home-based intervention in reducing caregiver burden, promoting caregiver mental health and reducing behavioural problems in elderly persons with dementia	Randomized controlled trial (RCT)	Assessments of caregivers: mental health by GHQ, caregiver burden by ZBS, distress due to behavioral disturbances by NPI-D, behavioral problems in the subject by NPI-S and ADL in the elder with dementia by EASI	Dyads of caregivers and PwD Caregivers—mean age (53)	Sample from the community in western India/Goa (Rural area)	In all, 73% dyads completed the trial. The intervention significantly reduced GHQ and NPI-D scores, with non-significant reductions in ZBS, EASI and NPI-S scores. Locally available, low-cost, home-based support is feasible and acceptable for caregivers of PwD, and significantly improve their mental health and care burden
Emmatty et al., 2006, [44]	The experience of burden in India: A study of dementia caregivers	To examine the relationship between caring for an elderly and caregivers’ experience of burden	Quantitative Cross sectional	Burden was assessed by ZBI. Open-ended questions were asked to map caregivers’ relationship to dementia patient and their caregiving experiences	Caregivers of PwD (*N* = 30), mean age (45)	Geriatric outpatient clinic of a tertiary hospital in southern India/Bengaluru (Urban area)	Only 30% of cases were in the high burden group. No significant correlation was found between caregiver relationship and burden scores. Extended families experienced less burden than other family types
Grover et al., 2017, [45]	Positive Aspects of Caregiving Experience among Caregivers of Patients with Dementia	To assess the positive aspects of caregiving and its correlates among caregivers of patients with dementia	Quantitative Cross sectional	SPACE, Coping Checklist, Social Support Questionnaire, WHOQOL-BREF version were used to assess positive aspects of caregiving. Burden Interview Schedule were used to assess caregiver burden	Dyads of primary caregivers and PwD (*N* = 55), mean age (information not provided)	Psychiatry outpatient of a multispecialty tertiary care hospital in northern India/Chandigarh (Urban area)	The highest mean SPACE scores were for caregiving motivation (2.63), caregiver satisfaction (2.54), and personal gains (2.4). More educated caregivers had significantly lower self-esteem and social caring scores. Greater burden correlated with lower caregiving motivation. Positive caregiving was linked to strong social support, being married, and higher avoidance coping use. Caregiver satisfaction improved their quality of life
Gurukartick et al., 2016, [46]	Social Determinants of Dementia and Caregivers’Perspectives in the Field Practice Villages of Rural Health Training Centre, Thiruvennainallur	To explore the family caregivers' perceptions and their support needs	An exploratory mixed-methods study design, where a qualitative method (key informant interview) was followed by a quantitative method (survey)	Vellore Screening Instrument for Dementia‑Informant version (VSID‑I ‑ Tool 2) was used to investigate family caregivers’ perceptions and their support needs	A representative sample of caregivers of PwD (*N* = 1300), mean age (71)	Rural health training center, department of community medicine of a medical college and hospital in southern India/Puducherry (Rural area)	Caregiving impacts caregivers’ personal and professional lives but caregiving is an integral part of Indian culture, and elderly prefer home‑based care. Caregivers preferred government owned public health facility for medical care. However, caregivers feel they lack adequate training
Hossien et al., 2017, [47]	Pathways to care among persons with dementia: Study from a tertiary care center	To explore the pathways taken by caregivers of dementia enroute to a tertiary care center and the interactions they had with different health care providers	Quantitative Cross-sectional	Semi-structured questionnaire. The Short Explanatory Model Interview (SEMI)	Purposive sample of caregivers of PwD (*N* = 35), mean age (39)	Geriatric clinic at a tertiary hospital in southern India/Bengaluru (Urban area)	Three pathways were identified: I) Neuropsychiatric: consulting a psychiatrist or a neurologist as first contact. 2) General Practitioner and 3) Non-cohesive: seeking various care options due to dissatisfaction. Overall, the caregivers lack information about dementia and its prognosis
Hurzuk et al., 2022, [16]	Understanding, experiences and attitudes of dementia in India: A qualitative study	To understand attitudes and perceptions concerning people with dementia residing in India in two metropolitan cities	Qualitative	Focus group discussions	A total of 58 participants took part in six focus group discussions and two individual interviews were conducted with persons with dementia	Alzheimer’s and Related Disorders Society of India (ARDSI) – a national organization which was the primary gatekeeper in recruiting participants for the focus group discussions. Participants were recruited separately in two metropolitan cities – Chennai (southernmost metropolitan city of India) and Delhi (North India) (Urban area)	Data from the focus group discussions and interviews revealed three overachieving themes: (1) Poor awareness, (2) Stigma and (3) Barriers to accessing care. All themes occurred within the context of socio-cultural aspects which typically framed discussions
Kazhungil et al., 2016, [48]	A comparative study of caregiver burden in late-onset depression and Alzheimer’s disease	To compare the caregiver burden in Late-onset depression (LOD) and AD and to identify factors associated with caregiver burden in LOD	Quantitative Cross sectional	Caregiver burden was assessed by the ZBI	Dyads of caregivers and patients with LOD with major depression (*N* = 25) mean age (47, 67, respectively). Dyads of caregivers and patients with AD (*N* = 25). Mean age (56, 71 respectively)	Medical college hospital, southern India/Manjeri (Rural area)	LOD and AD caregiving burdens are similar. Caregiver burden in AD is predicted by the Behavioral Pathology in Alzheimer’s Disease Scale score, lower income, presence of diabetes, and in-laws as caregivers
Lamech et al., 2019, [34]	The support needs of family caregivers of persons with dementia in India: Implications for health services	To explore the needs and challenges of family caregivers in Chennai, India	Qualitative	Focus group discussions and in-depth interviews were conducted using a topic guide	Purposive sample of family caregivers of PwD (*N* = 19), mean age (52)	Dementia care unit in SCARF (DEMCARES) out-patient facility in southern India/ Chennai (Urban area)	The themes highlighted caregivers’ challenges accessing treatment. Identified needs included person-centered care, trained health workers, information on dementia, advanced care needs, and cost-effective services
Lamech et al., 2021, [33]	Support groups for family caregivers of persons with dementia in India	To evaluate a support group service for caregivers of PwD; to identify the needs met and to explore the key facilitating factors and barriers for the caregivers to participate in the support group	Qualitative, prospective design	Observations and 11 group discussions conducted over one year	Convenience sample of caregivers attending an on-going support group (*N* = 22), mean age (56,5)	Dementia care unit in SCARF (DEMCARES) out-patient facility in southern India/Chennai (Urban area)	The support group met caregivers’ information, emotional, and counselling needs, with trust as a key facilitator. Barriers to participation included lack of home support, distance from the venue and work commitments. Support groups provide valuable information and peer support
Martis et al., 2022, [28]	Caring burden and quality of life among the caregivers of people living with dementia – a cross-sectional study in Udupi district of Karnataka	To assess the caring burden and QoL among the caregivers of people with dementia, as well as to ascertain the relationship between QoL scores and burden	Quantitative, Descriptive Cross-sectional study	The WHOQOL BREF questionnaire, which has 26 items was used to measure the caregivers’ overall QoL	Caregivers of PLWD (*N* = 80) with most of the participants (*N* = 53) living in rural area Average age 46 ± 12.7	Selected hospitals in the Udupi District (Rural area)	The majority of the participants (73.8%) experienced severe profound burden, while 17.5% experienced moderate to severe burden. The majority (*n* = 59, 73.8%) of the caregivers experienced severe profound burden, while few (*n* = 14, 17.5%) caregivers experienced moderate to severe burden. Results demonstrate that there is a significant association between the caregivers’ QoL, and the hours of care given per day to the PLWD
Narayan et al., 2015, [49]	Caregiving experiences of family members of persons with dementia in South India	To investigate to which extent the meaning and impact of caregiving are the same across cultures before adapting a family caregiving program model developed in USA	Qualitative	Semi structured interviews with caregivers of PwD based on an interview guide	FCGs of PwD (*N* = 30), mean age (51)	Geropsychiatric clinic at a tertiary hospital in southern India/Bengaluru (Urban area)	Family members knew “caregiving” but could not translate it, contrasting with the West. Dementia understanding was limited. FCGs reported feeling distressed, overwhelmed, and frustrated with caregiving. They were interested in an educational program, but had unrealistic expectations, including reversing the condition. The findings guide caregiver curriculum adaptations for Indian PwD caregivers
Natarajan et al., 2022, [27]	Acceptability of Social Robots and Adaptation of Hybrid-Face Robot for Dementia Care in India: A Qualitative Study	To understand the acceptability of social robots and adaptation of the hybrid face robot for dementia care in India	Qualitative	One focus group discussion with caregivers and professionals. In-depth interviews with the dyads	Dyads of PwD and their caregivers (*N* = 7), health care professionals (*N* = 2), technical experts in robotics (*N* = 2), mean age (information not provided)	Dementia care unit in SCARF (DEMCARES) out-patient facility in southern India/ Chennai (Urban area)	Three themes emerged: a) Acceptability of robots in dementia Care in India, b) Adaptation of Hybrid-Face robot and c) Future of robots in dementia Care. Caregivers and PwD welcomed social robots in dementia care, viewing them as helpful with caregiving challenges and foreseeing a positive future with robots
Pandya, 2019, [50]	Meditation Program Enhances Self-efficacy and Resilience of Home-based Caregivers of Older Adults with Alzheimer's: A Five-year Follow-up Study in Two South Asian Cities	To investigate the impact of a customized meditation program on caregiver burden mitigation and enhancing caregiving self-efficacy and resilience (as core outcomes) of home-based caregivers in Mumbai, India and Kathmandu, Nepal	Quantitative A 5-year follow-up, longitudinal study	ZBI, Revised Caregiving Self-Efficacy Scale, Resilience Scale for Adults and Caregiver Resilience Scale	Family caregivers of PwD. Mumbai: Control group; pre-/posttest (*N* = 56/43; intervention group pre /posttest (*N* = 58/51). Kathmandu; control group; pre/posttest (*N* = 33/24), intervention group; pre/posttest (*N* = 38/27). Mean age: control pretest and intervention pretest (53), Control post-test and intervention posttest (58)	Home-based caregivers, recruited through network of voluntary agencies working with older adults, geriatric clinics and units in private hospitals in Mumbai and Kathmandu (Urban area)	The intervention group reported less perceived caregiving burden and higher self-efficacy than the control group. Home practice significantly reduced caregiving burden. The culturally appropriate meditation program, drawing on familiar traditions, proved effective for home-based caregivers of Alzheimer’s. Women and spouse caregivers responded better to the program than male caregivers
Parveen et al., 2022, [51]	Knowledge and attitude of caregivers of people with dementia	To evaluate the knowledge and attitude of caregivers of people with dementia towards the Alzheimer disease, and to assess the association of attitude and knowledge towards dementia	Quantitative Cross-sectional	ADKS and Dementia Attitude Scale	Dyads of caregivers of PwD (*N* = 50), mean age caregivers (48)	Patient population attending psychogeriatric clinic outpatient services at a tertiary care hospital in northern India/Chandigarh (Urban area)	Most caregivers were aware of different aspects of dementia. The highest scores were for the course and symptoms, then “treatment and management.” The Dementia Attitude Scale’s mean score was 76.4 ± 18.4, indicating that caregivers hold fewer positive attitudes towards dementia
Pattanayak et al., 2010, [52]	Assessment of burden in caregivers of Alzheimer's disease from India	To assess the burden in relation to key variables and explore its predictors in caregivers of Alzheimer’s disease	Quantitative Cross- sectional	BAS	Dyads of caregivers and PwD (*N* = 32), mean age caregivers (54)	Dementia clinic of a tertiary hospital in northern India/New Delhi (Urban area)	Caregivers experienced moderate burden, increasing with patient’s behavior and cognitive impairment. Male patients’ caregivers felt more burdened than those for females. Female and older caregivers reported more physical, mental, and spouse-related burden. Caregivers from joint families had similar burden levels to nuclear families, except for less external support
Pattanayak et al., 2011, [53]	Coping and its relationship to quality of life in dementia caregivers	To assess coping strategies and Quality of life (QoL) and to studying the relationship between coping, QoL and severity of dementia	Quantitative Cross-sectional	Coping checklist (CCL) and WHOQOL-BREF Hindi version	Dyads of caregivers of PwD (*N* = 32), mean age caregivers (54)	Dementia clinic of a tertiary hospital in northern India/New Delhi (Urban area)	Two articles used the same dataset. Education positively influenced total coping score, problem-solving, positive distraction, acceptance, and negatively affected religion and denial. Problem-solving positively correlated with psychological QoL, while denial/blame negatively correlated with physical and psychological QoL. Social support, as the most beneficial coping strategy, positively influenced psychological, social, and environmental QoL domains. Coping strategies and QoL relied more on caregiver traits than patient’s dementia severity
Shaji et al., 2003, [54]	Caregivers of people with Alzheimer's disease: a qualitative study from the Indian 10/66 Dementia Research Network	To explore the care arrangements for people with dementia and the strain experienced by their family caregivers	Qualitative	Open ended individual interviews using a topic-based guide	Caregivers of PwD (*N* = 17), mean age (information not provided)	A rural community-based dementia care service in southern India/Thrissur (Rural area)	Most caregivers were young women, often daughters-in-law to dementia patients. Behavioral problems of dementia and incontinence caused strain, worsened by insufficient local health services and occasional family criticism. Family conflict was common. Many caregivers had significant mental health decline. Tragically, one caregiver committed suicide after her husband’s death
Shaji et al., 2009, [55]	Behavioral symptoms and caregiver burden in dementia	To examine the prevalence of BPSD in a community sample of patients with dementia and its impact on the caregivers	Quantitative Cross-sectional	Caregiver- Rated BEHAVE-AD, General Health Questionnaire (GHQ-12) was used to measure psychiatric morbidity. Caregiver strain was assessed by ZBI	Dyads of patients diagnosed with dementia and their caregivers (*N* = 29), mean age caregivers (information not provided)	Dementia care service in a rural community in southern India/Thrissur (Rural area)	Of 28 patients (96.6%), all had at least one BPSD as per BEHAVE -AD. A significant association existed between BEHAVE-AD’s total score and high caregiver distress on global rating. Caregiver burden negatively impacted the carer’s mental health
Sinha et al., 2017, [56]	Caregiver burden in Alzheimer-type dementia and psychosis: A comparative study from India	To evaluate the burden of care in caregivers of patients with Alzheimer-type dementia and compare it with elderly psychosis; and to also study the factors that influence burden of care in Alzheimer’s dementia	Quantitative Cross-sectional	NPI and ZBI were used to evaluate burden of care and to study the factors that influence burden of care	Dyads of caregiver-patients of Alzheimer-type dementia (*N* = 32), mean age (53) were compared with dyads of caregiver-elderly patients of psychosis (*N* = 32), mean age (50)	Outpatient departments of Psychiatry and Neurology at a tertiary center in northern India/Delhi (Urban area)	The mean dementia caregiver burden was 47.7, while elderly psychosis caregivers scored 33.6 (*p* < 0.001). Spouses bore the most burden, which in dementia was linked to cognitive impairment and ADL dysfunction. Caregivers’ psychological distress also indicated higher dementia burden
Tomita et al., 2010, [36]	Characteristics and Perceived Supports of Primary Caregivers of Home-Based Older Adults With Dementia in India, Taiwan, and the United States	To compare the characteristics of caregivers of older patients with dementia and describe their perception of social supports in India, Taiwan and the United States	Quantitative Cross-sectional, cross-national study	Interview form survey translated from English into Indian and Mandarin was conducted through person-to-person, phone and email. GDS was used to compare caregivers’ characteristics	Primary caregivers of home-based older adults with dementia; from India (*N* = 50) mean age (49), Taiwan (*N* = 67), mean age (54) and United States (*N* = 50), mean age (62)	Caregivers of PwD living in urban community recruited through psychiatric hospitals, day care center, private psychiatric clinics Alzheimer’s disease association’s website (Taiwan), Alzheimer’s clinics (United States), and (Mumbai, India – Urban area)	India, Taiwan, and the United States, despite their varied economies, healthcare systems, and cultures, rely greatly on informal caregivers for care of dementia patients. Differences were found in managing problematic behaviors. All Indian caregivers reported preventing wandering and unsafe behaviors, compared to 60% of Taiwanese and 50% of American caregivers. Universal and country-specific interventions were identified to alleviate caregivers’ burden
Tomita et al., 2010, [35]	Psychological Health of Primary Caregivers of Home-Based Older Adults with Dementia in India, Taiwan, and the United States	To identify positive and negative aspects of caregivers’ psychological health in the 3 countries	Quantitative Cross-sectional, cross-national study	ZBI, Center for Epidemiologic Studies Depression Scale (CES-D), UCLA Loneliness Scale and Picot Caregiver Rewards Scale were used to identify positive and negative aspects of caregivers’ psychological health	Primary caregivers of home-based older adults with dementia; from India (*N* = 50) mean age (49), Taiwan (*N* = 67), mean age (54) and United States (*N* = 50), mean age (62)	Caregivers of PwD living in urban community recruited through psychiatric hospitals, day care center, private psychiatric clinics Alzheimer’s disease association’s website (Taiwan), Alzheimer’s clinics (United States), and (Mumbai, India – Urban area)	Caregiver burden is closely associated with depression in India, Taiwan, and the US, and also correlates with loneliness. Indian caregivers reported the least negative psychological impacts, while Taiwanese caregivers experienced the worst outcomes of burden, depression, and severe loneliness. Unique predictors for psychological health were identified in each country. When accounting for caregivers’ residence in India, education about caregivers’ needs and governmental financial aid were deemed as essential interventions
Trivedi et al., 2013, [57]	Neuropsychiatric symptoms in mild cognitive impairment: An analysis and its impact on caregiving	To study the relevance of neuropsychiatric symptoms of MCI and the impact it has on caregivers of these patients	Quantitative, Cross-sectional consecutive sampling	GDS and NPI were used to assess NPS impact on caregivers	Total 90 dyads; 30 dyads of elderly-relatives in each of three groups. Group 1; dyads of relatives and their elderly with no cognitive problems (*N* = 30). Group 2; dyads of relative of patients with diagnosis of MCI (*N* = 30) Group 3: dyads of relatives and patients with dementia (*N* = 30), mean age of relatives (61, 63 and 70, respectively)	Psychiatry outpatient department of a tertiary care teaching municipal institute in western India, Mumbai (Urban area)	Neuropsychiatric complaints were reported by 73.33% of MCI group subjects, 90% of dementia group subjects, and 53.33% of normal cognition subjects. Distress was experienced by 73.33% of MCI group relatives, 90% of dementia group relatives, and 46.67% of control group relatives. Neuropsychiatric symptoms, increasing in frequency and severity with cognitive decline, differed significantly among the three groups and significantly predicted caregivers’ distress
Vayalil et al., 2015, [58]	A Qualitative Study on Lived Experience of Caregivers of Alzheimer’s Disease Clients and Effectiveness of the Booklet on Caregiver’s Burden at Selected Alzheimer’s Care Centers in Kerala	To assess the effectiveness of the use of Booklet “They are valuable for us” on caregivers’ burden	A triangulation approach to conduct the study. A quantitative approach was used to assess the care burden of caregivers of Alzheimer’s disease clients and qualitative approach using the phenomenological method was adopted to qualitatively analyse the care burden	ZBI was used to assess the effectiveness of the Booklet on caregivers’ burden. Semi-structured interview to explore the lived experience of care givers of Alzheimer’s disease client	Caregivers of PwD (*N* = 40), mean age (information not provided)	Alzheimer’s care center in southern India/Kottayam (Urban area)	The mean pretest care burden was 37.2, decreasing to 30.1 post-test (*p* < 0.05). The study found the booklet “They are valuable for us” effectively reduced caregivers’ burden of Alzheimer’s patients

*AD* Alzheimer’s Disease, *ADKS* Alzheimer’s Disease Knowledge Scale, *ADL* Activities of Daily Living, *ARDSI* Alzheimer’s Related Disorders Society of India, *BAS* Burden Assessment Schedule, *BPSD* Behavioral and Psychological Symptoms of Dementia, *CCL* Coping Checklist, *CDR* Clinical Dementia Rating scale, *EASI* Everyday Abilities Scale for India, *FCGs* Family caregivers, *GDS* Global Deterioration Scale, *GHQ* General Health Questionnaire, *HCP* Health Care Personnel, *MCI* Mild Cognitive Impairment, *MMSE* Mini-Mental State Examination, *NPI* Neuropsychiatric Inventory, *NPI-D* Neuropsychiatric Inventory-Distress, *NPI-S* Neuropsychiatric Inventory-Subject, *NPS* Neuropsychiatric Symptoms, *PSS* Perceived Stress Scale, *PwD* People/Patient/Patients with Dementia, *RCT* Randomized Controlled Trial, *SPACE* Scale for Positive Aspects of Caregiving Experience, *VSID-I-Tool 2* Vellore Screening Instrument for Dementia-Informant Version, *WHOQOL-BREF* World Health Organization Quality of Life–BREF version, *ZBI* Zarit Caregiver Burden Interview, *ZBS* Zarit Burden Score, *ZCBS* Zarit Caregiver’s Burden Scale

The same four authors (ARG, HB, LH, and DL) synthesized the extracted data to achieve the aim of the review. The results from the quantitative studies are presented in a narrative summary to facilitate their integration with the findings from qualitative studies [59]. Key findings with common themes across studies were grouped under the same categories. Two other authors (PTS and SG) reviewed the manuscript draft and provided feedback.

## Results

Our search for studies on FCGs and healthcare personnel’s experiences, challenges, and needs in caring for PwD in India initially identified 611 publications through database searches. Additionally, two more publications were identified via email notifications resulting in 613 publications. After removing three duplicates, we screened 610 studies. Of these, 267 were excluded based on titles, and 303 were excluded after abstract reviews. We then proceeded to review 40 full-text studies, excluding 13 because they did not meet the inclusion criteria. Ultimately, the final sample consisted of 27 publications, including two additional studies identified during an updated search in September 2024.

### Study characteristics

Although all the included studies were conducted in India, they spanned various regions. The majority took place in urban centers such as Delhi, Mumbai, and Kolkata, and only six of 27 studies were conducted in rural areas across both northern and southern parts of the country [28, 43, 46, 48, 54, 55].

Of the 27 studies included, two had a mixed-methods design [46, 58], eight were qualitative [16, 27, 33, 34, 41, 42, 49, 54], and 17 were quantitative [26, 28, 35, 36, 40, 43–45, 47, 48, 50–53, 55–57]. The studies were published between 2003 and 2024. More than half of the studies were carried out in southern India. Overall, the studies’ samples consisted of FCGs (*N* = 2,707), FCGs in dyads with PwD (*N* = 490), and healthcare personnel (*N* = 12), including nurses, psychologists, social workers, a geriatrician, and a psychiatrist. Only data from FCGs and healthcare personnel’ perspectives were analyzed in this review.

Of the eight qualitative studies, three [16, 27, 34] that focused on PwD, FCGs, and healthcare personnel as participants employed both focus group interviews and individual interviews as data collection methods. One study employed only focus group interviews [41] with FCGs and healthcare personnel. While three studies [42, 49, 54] used only individual interviews with FCGs as a data collection method, one [33] conducted observations of a support group consisiting of FCGs. One study [27] also included two technical experts in robotics. Most studies performed purposeful sampling, and all studies provided information about participant recruitment. The individual or focus group interviews were conducted in Hindi and English or other regional languages depending on the study setting, and they lasted 30 – 60 min. The methods of analysis varied and included thematic analysis [16, 27, 33, 34, 41], constant comparative analysis [49, 54], and interpretative phenomenological analysis [42].

The studies with a mixed-methods design [46, 58] employed both qualitative (key informant interview) and quantitative method (survey). The qualitative data were analyzed using content analysis, while multiple logistic regression was applied to the quantitative data.

Among the quantitative studies, three were randomized controlled trials [40, 43, 50], 14 had a cross-sectional design [26, 28, 35, 36, 44, 45, 47, 48, 51–53, 55–57]. From the studies presenting results from comparing FCGs’ experiences in multiple countries [35, 36], only data presenting Indian caregivers’ experiences were extracted and analyzed.

In the quantitative studies, several instruments were administrated to the FCGs. For example, Anand et al. [40] used the Perceived Stress Scale to measure FCGs’ subjective perception of stress. Basu et al. [26] employed the Neuro-Psychiatric Inventory (NPI) to evaluate the relationship between the neuropsychiatric symptoms of dementia and caregiver burden. Dias et al. [43] adopted the Zarit Burden Scale, the General Health Questionnaire (GHQ), the NPI, and the Everyday Abilities Scale for India to evaluate the effectiveness of a home-based intervention to reduce caregiver burden. Emmatty et al. [44] used the Zarit Burden Interview (ZBI) to examine the relationship between being a caregiver of an elderly relative living with dementia and caregiver burden. Grover et al. [45] employed the Scale for Positive Aspects of Caregiving Experience, the Coping Checklist-Hindi Version, the Social Support Questionnaire, the World Health Organization Quality of Life-BREF (WHOQOL-BREF), and the Burden Interview Schedule to assess the positive aspects of caregiving and its correlates among caregivers of patients with dementia. Kazhungil et al. [48] adopted the ZBI to compare the caregiver burden in late-onset depression (LOD) and Alzheimer’s disease (AD). Martis et al. [28] used the WHOQOL-BREF and the ZBI to assess quality of life and caregiver burden among caregivers of PwD. Pandya et al. [50] employed the ZBI, the Revised Caregiving Self-Efficacy Scale, the Resilience Scale for Adults, and the Caregiver Resilience Scale to evaluate whether a meditation program enhances the self-efficacy and resilience of home-based caregivers for individuals with AD. Parveen et al. [51] adopted the Dementia Attitude Scale and the Alzheimer’s Disease Knowledge Scale to assess the level of knowledge and attitude among caregivers of PwD. Pattanayak et al. [53] used the Coping Checklist and the WHOQOL-BREF (Hindi) to assess coping strategies and quality of life of caregivers. Pattanayak et al. [52] employed the Burden Assessment Schedule to assess caregivers’ perceptions of caregiver burden. Shaji et al. [55] adopted the ZBI, the GHQ-12, and the Behavioral Symptoms in Alzheimer’s Disease to evaluate the impact of behavioral symptoms and caregiver burden in dementia. Sinha et al. [56] used the ZBI and GHQ-12 to evaluate the burden of care in caregivers of PwD. Tomita et al. [35] employed the ZBI, the Center for Epidemiological Studies Depression Scale, the University of California Los Angeles Loneliness Scale, and the Picot Caregiver Rewards Scale to assess psychological health of the primary caregivers of home-based older adults with dementia in India, Taiwan, and the US.

In their mixed-methods studies, Gurukartick et al. [46] adopted Vellore Screening Instrument for Dementia-Informant to investigate the FCGs’ perceptions and their support needs. Vayalil et al. [58] applied the Zarit Caregiver Burden Scale to evaluate the effectiveness of using a booklet prepared on care of the patient with AD, in alleviating caregivers’ care burden.

Among the studies included, six were intervention based. Two explored the acceptability of different programs, such as an online training and support program for the FCGs of PwD [41], and the implementation of social robots and the adaptation of a hybrid face robot for dementia care [27]. Three studies assessed the impact of specific programs, including a meditation program [50], support groups for FCGs [33], and a home care program aimed at supporting caregivers [43]. Lastly, one study [58] evaluated the effectiveness of a booklet prepared on care of the patient with AD in reducing caregiver burden. Table 1 provides detailed information on the characteristics of the studies included.

### Data synthesis and analysis

Considering the aim of the study, the analysis of the quantitative and qualitative data resulted in three major themes: (1) navigating the dual realities of caregiving: challenges and rewards for FCGs in India, (2) gaps in support for home-based care: challenges faced by FCGs in rural parts of India, and (3) addressing the present and future needs of caregivers for PwD in India*.* Table 2 presents an overview of the themes, with excerpts from the results or findings sections of the included articles:

**Table 2:** Themes and synthesized findings

Findings	Synthetized findings	Themes
The higher dependency of patients with Alzheimer’s disease (AD) or mild cognitive impairment (MCI) than patients with other chronic diseases on caregivers for activities of daily living (ADL) was associated with caregivers’ higher scores on the Perceived Stress Scale [40] Caregiver’s burden in providing dementia care positively correlated with their psychological distress and PwD’s cognitive impairment and inability to perform ADL functions [56] The burden of caregivers of PwD is not specific to India, but global [36] Family caregivers (FCGs) reported feeling distressed, overwhelmed, and frustrated with caregiving [49]	Caring for people with dementia (PwD) is associated with high degree of burden and perceived stress among FCGs	Navigating the dual realities of caregiving: challenges and rewards for FCGs of PwD in India
Caregivers of PwD with higher levels of behavioral and psychological symptoms (BPSD) reported higher levels of burden than caregivers of PwD with lower levels of BPSD. Caregivers generally reported physical and emotional burn-out [26] Poor quality of life arises from caring for a loved one who has dementia [28] Behavioral symptoms are common and cause significant distress to PwD and their caregivers [49], and they can predict caregiver burden in AD [48] Major sources of strain for caregivers were PwD’s behavioral problems and incontinence. Most caregivers reported significant deterioration in their mental health, and one committed suicide following their spouse’s death [54] The severity and frequency of neuropsychiatric symptoms (NPS) differed significantly between individuals with normal cognition, MCI and PwD and significantly predicted caregivers’ distress [57] PwD was generally isolated by the family, as they feared how others would react to the condition [16]	PwD’s behavioral problems are a major cause of caregivers’ stress, burden and stigma	
Caregivers of male (vs. female) patients reported a higher burden. The female (vs. male) caregivers perceived a higher burden on physical and mental health, and older caregivers perceived a higher burden from caregiving routine and spouse-related issues [52] The final change was that a positive aspect of caregiving was considered as an outcome rather than a secondary intrapsychic strain [35] … Caregiving as a process is not always associated with only negative consequences. Many caregivers do experience positive aspects of the caregiving experience [45]	Caregiving for PwD is associated with a high burden, especially when caring for male patients Perceived positive outcomes of caregiving	
In India, few people with dementia are seen by doctors [54] According to FCGs and healthcare personnel, lack of time, limited internet access, digital illiteracy and difficulty with reaching rural populations complicate using an online training and support program for dementia in India [41] … a huge population who access the Internet through different languages will be left out, especially in the semiurban and rural areas [46]	Challenges and barriers to accessing information and support are common	Gaps in support for home-based care: challenges faced by FCGs in rural parts of India
Caregivers reported using inappropriate coercive measures (e.g. sedatives, seclusion, environmental restraint, and restricted dietary intake) to protect PwD and manage their behavioral problems and ADL [42] There is a need to evaluate the attitude of FCGs of PwD from time to time and address prevailing negative attitude and support them while they are performing the caregiver role [51]	Caregivers use different coercive measures due to a lack of support with managing behavioral symptoms	
Lack of help at home to support PwD, distance from the venue and professional commitments prevented caregivers from participating in a support group [33] Lack of awareness about the existence of the program, difficulty in reaching out to the rural population, accessibility to a computer and Internet connectivity …, were reported as limitations [46]	Caregivers lack practical help at home	
Caregivers reported poor social lives and emotional well-being, lack of non-caring activities and job opportunities [41] Social support is the most beneficial coping strategy and was found to be positively correlated with psychological, social and environmental domains of quality of life [53]	Caregivers lack time for their personal lives and non-caring activities	
Caregivers were poorly informed about dementia and its prognosis [34] Family caregivers of PwD have less knowledge about the disease condition and they find it difficult to manage problems of toileting, bathing, anger and wandering. The information provided in the booklet was effective in reducing the care burden of caregivers of PwD [58] Per FCG and healthcare personnel, the online training and support program would benefit from using simple language, culturally relevant examples, and an interactive design [41] Although caregivers were interested in an educational program, many had unrealistic expectations (e.g. reversing the condition) [49] All caregivers strongly expressed being insufficiently trained in caregiving [47]	Information about dementia and feasible online training support programs are needed	Addressing the present and future needs of caregivers for PwD in India
Respite care and community support services are needed [43] Locally available, home-based support and low-cost human resources are feasible and accepted by caregivers of PwD and can significantly improve caregivers’ mental health and burden of care [45] Community-based interventions in dementia care might be particularly suitable to address barriers families face in rural areas [44] Caregivers preferred government-owned public health facilities for medical care [47] Person-centered care, cost-effective services, skilled healthcare workers and information on dementia and advanced care needs are needed [49]	Community support, home-based services and respite care can be provided by skilled and knowledgeable healthcare workers	
Stronger social support, being married and frequently using avoidance coping were associated with positive caregiving experiences [46] Social support was the most beneficial coping strategy and positively correlated with psychological, social and environmental domains of quality of life [35] A customized meditation program was culturally appropriate (e.g. drew on traditions familiar to participants) and an effective intervention for home-based caregivers of older adults with AD [50]	Culturally adapted coping strategies can combat stress and burden related to caregiving	
Caregivers and PwD were open to using a social robot in dementia care. Caregivers perceived that it would help with the challenges of caregiving and positively viewed a future with robots [27] Education about caregivers’ needs and governmental financial support were identified as necessary interventions in the future [35]	Openness to innovative interventions (e.g. using social robots in dementia care and digitalization), education for caregivers and financial support are needed	

### Navigating the dual realities of caregiving: challenges and rewards for FCGs in India

Several studies have addressed FCGs’ burden of caring for their family members with dementia, and some have reported interventions to reduce FCGs’ caregiving-related stress and burden. The studies showed the substantial burden and stress experienced by FCGs resulting from PwD’s increasing cognitive impairment and dependence on them for performing activities of daily life (ADL) [40, 42, 56], as well as from the behavioral and psychological symptoms of dementia (BPSD) [26, 55, 57]. The FCGs experienced physical and emotional burnout and deterioration in their mental health, thus affecting their personal and professional lives. The greater the caregiving burden, the lower the quality of life they perceived [28]. The caregivers of male patients reported a higher burden than the caregivers of female patients [52]. Moreover, because of poor awareness of dementia as a medical condition among Indian population, especially in the rural parts of India, healthcare workers reported families isolating PwD; they feared how others would react to the condition, stigmatizing the whole family [16]. Stigma is perceived as one of the many factors contributing to caregivers’ burden [54].

Three studies investigated the stress and burden among caregivers of PwD compared with that of caregivers of patients with other chronic diseases, including LOD [48], chronic medical and other psychiatric disorders [40], and psychosis in older adults [56]. These studies revealed that the caregivers of PwD experience greater burden and stress than the caregivers of patients with other chronic diseases.

Two multinational studies explored the similarities and differences in caregiving aspects among the caregivers of PwD in India, Taiwan, and the US [35, 36]. They found that a country’s economic development does not necessarily improve caregivers’ psychological health. Despite differences in economic development, healthcare systems, culture, and traditions, the three countries they examined depend heavily on FCGs to care for PwD in their homes and follow similar patterns in caregiving [35, 36]. However, although most of the studies revealed challenges with being FCG to family members with dementia, two studies [35, 45] revealed that caregiving is associated not only with only negative consequences but also with motivation and satisfaction in caregiving.

### Gaps in support for home-based care: challenges faced by FCGs in rural parts of India

Several studies have indicated that FCGs often face a lack of support, access to respite care, and adequate training in providing care to PwD [44, 46, 52, 58]. In some cases, FCGs report using inappropriate coercive measures, such as seclusion, sedatives, and environmental restraints, to safeguard PwD and manage their behavioral problems and ADL [42]. Family caregivers strongly believe that old people prefer home-based care, but they need information and guidance on how to provide care [46].

Some intervention studies have evaluated the effectiveness of home-based intervention programs in reducing caregivers’ stress and burden. Dias et al. [43] revealed that supporting caregivers by providing information about dementia via various modes, including information about assisting with ADL, managing BPSD, and following up with psychiatrists, significantly improved caregivers’ mental health and reduced the care burden compared to the control group. Pandya [50] developed a meditation-focused program for FCGs and conducted a five-year follow-up with the participants. The results showed that caregivers in the intervention group reported a reduced perceived caregiving burden and greater self-efficacy in obtaining respite care and managing upsetting behaviors and thoughts compared with caregivers in the control group. Caring for a loved one with dementia can often feel overwhelming, but with more information and support, FCGs can provide better care. Vayalil et al. [58] addressed this challenge by developing a booklet for FCGs containing information about AD and caregiving strategies. The booklet’s content was delivered to FCGs through a seminar conducted in two research settings. The results showed a significant reduction in caregivers’ burden after they used the booklet for one month [58].

From healthcare personnel’s perspectives, FCGs need information that is tailored specifically to each stage of dementia, along with support that affords them time for themselves without feeling guilty [16, 41]. A lack of awareness about the availability of programs or locally existing services for PwD, such as day-care centers, was also expressed by FCGs [41]. Insufficient time, digital illiteracy, poor internet access, and difficulty reaching rural population because of the lack of awareness about the internet and low levels of education were some of the challenges reported by healthcare personnel when providing online training and support programs for FCGs [41].

### Addressing the present and future needs of caregivers for PwD in India

Family caregivers reported that knowledge and awareness of dementia in India are poor and that they lack information about the illness as a medical condition and its prognosis. Therefore, many FCGs had unrealistic expectations of dementia care, including the possibility that the condition could be reversed [34, 49].

In several studies, FCGs expressed the need for respite care and locally available, low-cost, human-based support [43, 45]. They preferred government-owned public health facilities and care from skilled and knowledgeable healthcare personnel [47, 49]. Family caregivers also expressed a need for social support and help with culturally appropriate coping strategies based on traditions that are familiar to participants, including a customized meditation program to combat the stress and burden related to caregiving [35, 46, 50].

Healthcare personnel expressed that interactive online training programs for FCGs should use simply written, user-friendly materials supported by videos and web platforms that are compatible with mobile phones to enhance their utility [41]. Family caregivers and PwD were open to digitalized support and innovative ideas, including the use of social robots in dementia care [27], which FCGs and healthcare personnel perceived would be helpful in dealing with the challenges of caregiving, especially given projected needs for dementia care in the future.

Several studies emphasized the idea that FCGs and healthcare personnel need appropriate interventions, such as home healthcare education, awareness camps, self-help groups, and community support groups, to enhance awareness of dementia as a medical condition [28, 40]. There is also a need for educational support and training for both FCGs and healthcare professionals to better understand the use of coercive measures and their impact on PwD [42], especially in rural areas [28, 43, 46, 48, 54, 55]. This will contribute to the development of healthcare policy in the future [26], which will, in turn provide comprehensive care for PwD.

## Discussion

The aim of the current scoping review is to examine the existing literature on the experiences of FCGs and healthcare personnel involved in the care of PwD in India. To our knowledge, this scoping review is the first to focus on FCGs and healthcare personnel’s experiences, challenges, and needs in caring for PwD in India. The review, presenting evidence from 27 primary studies conducted in both rural and urban parts of India, offers important insights into FCGs’ and healthcare personnel’s challenges and needs when providing care to PwD in India. This could help raise awareness of dementia as a medical condition while addressing the needs of both FCGs and healthcare personnel in their caregiving roles.

Similar to findings reported in studies conducted in other parts of the world [60–62], our results show that dementia can lead to increased stress, stigma, and burden, all of which may negatively affect the mental and physical health of FCGs. The findings also indicate that, regardless of country, the burden of caregiving is commonly perceived by FCGs [35, 36]. Similar results were also obtain in a recently published scoping review that compare high-and low- to middle-income Asian countries [62]. Therefore, WHO launched the global action plan on the public response to dementia 2017 – 2025 [63], signaling an important step forward in achieving physical, mental, and social well-being for PwD, their FCGs, and healthcare personnel. The report shows that while some progress is being made, urgent increased efforts are needed globally to reach dementia targets by 2025.

Our results revealed a higher perceived stress among the FCGs of male PwD than among the FCGs of female PwD [52] and that spouses are more likely to be FCGs for a male PwD while female PwD are more likely to be cared for by their children or children-in-law in addition to their spouses. This finding is grounded in Indian people’s cultural background and family values, in which caring for the elderly is considered the responsibility of immediate relatives, such as children or children-in-law [64]. Although caring of older adults, including those with cognitive impairment, is an important family task in India’s culture and traditional joint family system [64], the stigma of being FCGs for PwD is also experienced among family members. Recent research has demonstrated that Indian male caregivers experience greater stigma than female caregivers [65], as the role of caregiving is mainly attributed to females. Male caregivers prefer approaching a close friend or another family member to seek help, but very few seek professional help, as this may have negative social impact on FCGs. This highlights a lack of adequate knowledge and awareness about seeking professional help [54], particularly in rural areas where caregiving is motivated by a sense of marital duty or filial responsibility [49] or is constrained by a low socioeconomic background [34].

The role of Indian culture, in which myths and misconceptions play a key role in shaping one’s perceptions about dementia, also reduces the desire for seeking professional help when required. Furthermore, it may suggest that individuals are concerned about embarrassment and the loss of honor for male caregivers, which could result in social exclusion, or missed opportunities for female caregivers, particularly daughters, to get married [66]. However, the current scoping review reveals that FCGs are usually insufficiently equipped to assume a caregiving role. As India continues to experience the disintegration of the joint family system and as individuals increasingly embrace modern lifestyles, social dynamics, economic structures, and cultural norms have changed significantly [67]. Therefore, cases in which FCGs use coercive measures to safeguard family members with dementia and assist them with ADL have been reported [42], and these abuse-prone practices may increase without an appropriate support system for the caregivers of PwD. However, similar to the findings from a previous study conducted in an Indian context [68], our results revealed that not all caregiving is perceived as a burden [45]. Caregiving for PwD can be a positive experience for FCGs, which not only contributes to their personal and spiritual growth but also provides motivation and satisfaction in caregiving, thus strengthens family bonds [35, 45].

The results from the current review have shown that FCGs in India need person-centered, cost-effective social support, preferably government-owned public health and respite care services that are home based and culturally accepted [43, 49, 50]. For example, results from Pandya’s study [50] reveal that Hindu caregivers responded better to the meditation program than Buddhist caregivers, suggesting that the program needs to be refined for Buddhist participants, which points to the importance of cultural differences in cultural differences in tailoring interventions to meet the specific needs of diverse groups. Even so, the needs expressed by caregivers in India are quite similar to those of caregivers of PwD elsewhere in the world [62, 69]. Our results also indicate that even among healthcare personnel, there is a common impression that dementia care is a specialized service and that only experts can provide such care, which may be lacking in rural areas [16]. There is little knowledge and awareness of dementia in India, especially among FCGs from rural areas who lack information about the prognosis of this condition and strongly express the need for adequate information and training in caring for PwD at home [34, 49]. However, FCGs’ lack of knowledge about dementia could stem from a lack of related health services and knowledge among healthcare personnel. The results of a study [21] demonstrated that knowledge about dementia is limited among nursing staff and other healthcare personnel in India. These results are similar to those of studies conducted in other parts of the world, indicating that PwD and their FCGs have limited knowledge, and that healthcare personnel need knowledge, skills, and confidence to meet the needs of PwD and their FCGs [70, 71].

Our review additionally demonstrates some disparities in dementia healthcare services between urban and rural areas in different parts of India. Similar to another systematic review [72], we found that the challenges of rural living included difficulties reaching dementia care services and the detrimental aspects of living in a rural community, such as social isolation, transportation challenges, and limited professional caregivers. Disparities across many domains, especially the higher mortality rates in PwD and their caregivers who live in rural areas compared with those who live in urban areas [43], were also featured in another systematic review [73]. According to WHO [74], the health disparities experienced by rural populations result from adverse social and environmental determinants and the poor health systems in rural areas. Several international studies suggest the importance of innovative technological solutions for empowering and supporting PwD and their FCGs [75–77]. Although India’s economy is growing well, its rural infrastructure is improving, and people have access to technology [78], digital illiteracy, poor internet access, and difficulties in reaching rural population remain major challenges in using online training and support programs [79]. These issues contribute to the reduced use of healthcare services.

Despite the government of India launching the flagship Digital India Campaign in 2015, which included public health initiatives aimed at leveraging digital technologies to enhance healthcare access in rural areas, significant barriers continue to hinder the successful implementation of digital transformation [80]. Some of these barriers include limited network coverage, inadequate information technology infrastructure, high installation and operational costs, absence of comprehensive medical records and qualified experts, and user-related challenges. Although new digital technologies, such as the hybrid-face robots [27], have begun to make their way into India, there is an urgent need for culturally tailored, relevant, and easy-to-understand online caregiver support programs. Overcoming these barriers requires partnership among governments, nongovernmental organizations (NGOs), healthcare professionals, patients, and their caregivers [80].

Technological solutions present a promising means to assist FCGs in seeking information to improve the care that they provide to PwD. As our review suggests, the information provided by the digital and web-based educational resources currently available in India is not always easily accessible or adequately tailored to the FCGs’ diverse needs. Given the growing number of PwD in India, a wide gap exists between future needs for support and respite care and the availability of day care centers and full-time care facilities suitable for PwD [81]. The number of such centers and facilities is increasing, especially in large cities, but at a rather slow pace [82]. Although India is one of the fastest-developing countries worldwide, a large proportion of families face basic health problems and challenges in accessing healthcare services and information, including supportive health and welfare services for PwD and their FCGs [83].

The current Indian care services for dementia are vastly inadequate [81]. India is a diverse country with a multitude of languages and dialects and vastly varying educational levels among its inhabitants. Broadly, Indian society can be categorized into urban and rural India. Urban India includes the urban poor, such as migrant workers from rural areas, who typically have low literacy rates, as well as the middle class, who generally have high literacy levels and good access to healthcare. By contrast, rural India is primarily made up of agriculture-dependent populations residing in villages, often from lower socioeconomic backgrounds than their urban counterparts [84]. This rural population tends to have low health literacy rates and limited access to healthcare services. Therefore, as the findings from our review suggest, healthcare personnel need to have adequate knowledge about dementia to ensure the quality of care provided to PwD and to empower FCGs with knowledge and guidance; in this way, they can cope positively with their responsibilities, minimizing caregiving’s negative impacts on their health and alleviating their burden as caregivers [16, 27, 34, 41].

Interventions to empower the FCGs of PwD, such as meditation programs [50], can facilitate a positive caregiving experience, a sense of satisfaction with caregiving, and the opportunity to give back to their loved ones, which is deeply rooted in Indian culture [45]. Although NGOs, such as Alzheimer’s and Related Disorders Society of India and Dementia India Alliance [16, 41], provide information, healthcare services, and social support for PwD and their FCGs via day care centers, memory clinics, dementia help lines, group meetings, and their websites (ardsi.org and dementia-india.org., respectively) are located only in a few large cities, thereby leaving out rural regions of India from availing such services. The geographical distance from rural to urban areas to use adequate healthcare services imposes an additional financial burden because of logistical expenses and increased financial resource use by FCGs, further exacerbating the already considerable caregiver burden. However, these programs are essential for providing dementia-related information to caregivers in rural regions of India, where professional, in-person support is not readily accessible [41]. Altogether, access to respite care, online information, and training programs could meet some of the future needs for dementia care in India.

### Strengths and limitations

To the best of our knowledge, this study is the first to provide a comprehensive overview of the experiences of FCGs and healthcare personnel in providing care to PwD in India. While a key strength of our review is its specific focus on India, this could also be seen as a limitation. The publications included in this scoping review were limited to studies conducted in India, a context that has not been previously explored in relation to caregiving for individuals with dementia. As a result, the findings from our review are primarily applicable to the Indian healthcare context and may have limited transferability to other settings. However, because of India’s notable cultural and socioeconomic similarities to other Asian countries, the results of this scoping review may be applicable and relevant to similar contexts across the Asian continent.

The included studies feature diverse objectives, designs, and data collection methods, covering various Indian contexts from both urban and rural areas, and all are peer reviewed, which enhances the strength of the review. Additionally, the scoping review adhered to the guidelines outlined in the JBI Manual, with at least two researchers involved in each step of the review process. This enhanced the review process and the practical validation of the results, thereby improving the overall reliability of the study.

A limitation that should be mentioned is that we did not include grey literature, which may have led to the omission of relevant publications. Additionally, our review might have overlooked studies not published in English or those not indexed in the databases we searched. To ensure accessibility for an international audience, we deliberately excluded studies published in Indian journals and those written in regional Indian languages. Nevertheless, within the range of the databases searched, efforts were made to encompass all pertinent publications based on the established inclusion and exclusion criteria.

Some studies included in the current review have a small number of healthcare personnel as participants who reported their experiences with dementia care. Although the exact reason for few studies including healthcare personnel as participants remains unclear, a possible reason is that their involvement in providing direct patient care is limited in the Indian context compared with developed countries. In India, family caregiving is a priority, while in developed countries, healthcare services provide direct patient care in home-based care or long-term care facilities. An additional consideration is the underrepresentation of studies conducted in rural areas of India. This disparity may stem from the relatively easier access to participants and better research facilities in urban settings, as well as the prevailing perception that urban issues are more relevant or pressing for academic and policy-oriented research.

## Recommendations

Family caregiving is a fundamental aspect of dementia care in India and is likely to remain so despite facing various emerging challenges and demographic shifts. Future research on dementia care in India should emphasize recognizing FCGs as integral partners, increasing awareness about dementia and its consequences for PwD, their family, and community, and developing intervention programs that address these challenges. Programs should be designed to build skills for both FCGs and healthcare personnel and be adapted to the specific needs and cultural backgrounds of PwD and their caregivers from both urban and rural areas in India. Moving forward, supporting and strengthening the FCGs of PwD in India will involve identifying effective solutions within the constraints and opportunities presented by demographic changes and cultural diversity. Prioritizing the use of local rural resources can help develop cost-effective, sustainable dementia care services for PwD and their FCGs in communities. Free or government-subsidized rural healthcare services, along with education and training programs for healthcare personnel, can further motivate their participation in delivering adequate healthcare services to PwD and their caregivers in rural communities across India.

## Conclusion

The literature on FCGs’ and healthcare personnel’s experiences in supporting PwD in India remains limited but is growing. This scoping review highlights the results of studies that explored the experiences, challenges, and needs of FCGs and healthcare personnel involved in dementia care across various regions in India, with a concentration in Southern India. The challenges identified include perceived caregiver burden, stress, and poor health among FCGs, who often lack adequate support, respite care, and sufficient information about dementia. As India’s older population rapidly increases because of long life expectancies, the prevalence of dementia-related health issues is also rising. There is an urgent need for culturally relevant, person-centered, and cost-effective educational and support programs, especially in India’s rural areas, to address these current and future needs. Further research is also needed to explore the experiences of healthcare personnel in dementia care to enhance the overall quality of support for PwD and their caregivers in rural areas of India.

## Supplementary Information

Supplementary Material 1.

Supplementary Material 2.

## Data Availability

The authors confirm that all data generated or analyzed during this study are included in this published article. Furthermore, primary and secondary sources and data supporting the findings of this study were all publicly available at the time of submission (Table 1).

## Abbreviations

AD: Alzheimer’s disease
ADL: Activities of daily living
BPSD: Behavioral and psychological symptoms
FCGs: Family caregivers
GHQ: General health questionnaire
GHQ-12: The 12-items general health questionnaire
LOD: Late-onset dementia
NPI: Neuro-psychiatric inventory
PwD: People with dementia
WHOQOL-BREEF: World Health Organization quality of life – brief
