# Supplementary material for: Caregivers’ experiences, challenges, and needs in caring for people with dementia in India: a scoping review
Source: BMC Health Serv Res. 2024 Dec 30;24:1661. doi: 10.1186/s12913-024-12146-x (PMC11684304; doi:10.1186/s12913-024-12146-x)
Supplement: Supplementary file 1 — Supplementary Material 1. [file 12913_2024_12146_MOESM1_ESM.docx]

**An example of a recent data search in the Medline/Ovid database per September 4, 2024**

1 dementia/ or alzheimer disease/ or dementia, vascular/ or dementia, multi-infarct/ or frontotemporal lobar degeneration/ or frontotemporal dementia/ or "pick disease of the brain"/ or lewy body disease/ 96437

2 (dementia or alzheimer*).tw,kw,kf. 0

3 1 or 2 96437

4 Caregivers/ 40101

5 health personnel/ or allied health personnel/ or nursing assistants/ or psychiatric aides/ or exp medical staff/ or exp nurses/ or exp nursing staff/ 64726

6 (health care personnel or nurse* or doctor* or physician* or health care professional* or health care worker*).tw,kw,kf. 0

7 (caregiver* or family or relative* or spouse* or families).tw,kw,kf. 0

8 4 or 5 or 6 or 7 103193

9 India/ 7805

10 (india or indian population*).tw,kw,kf. 0

11 india.in,cp. 42417

12 9 or 10 or 11 50211

13 3 and 8 and 12 51

14 (experience* or perspective* or living).tw,kw,kf. 0

15 13 and 14 0

16 dementia/ or alzheimer disease/ or dementia, vascular/ or dementia, multi-infarct/ or frontotemporal lobar degeneration/ or frontotemporal dementia/ or "pick disease of the brain"/ or lewy body disease/ 96437

17 (dementia or alzheimer*).tw,kw,kf. 0

18 16 or 17 96437

19 Caregivers/ 40101

20 health personnel/ or allied health personnel/ or nursing assistants/ or psychiatric aides/ or exp medical staff/ or exp nurses/ or exp nursing staff/ 64726

21 (health care personnel or nurse* or doctor* or physician* or health care professional* or health care worker*).tw,kw,kf. 0

22 (caregiver* or family or relative* or spouse* or families).tw,kw,kf. 0

23 19 or 20 or 21 or 22 103193

24 India/ 7805

25 (india or indian population*).tw,kw,kf. 0

26 india.in,cp. 42417

27 24 or 25 or 26 50211

28 18 and 23 and 27 51

29 (experience* or perspective* or living).tw,kw,kf. 0

30 28 and 29 0

31 dementia/ or alzheimer disease/ or dementia, vascular/ or dementia, multi-infarct/ or frontotemporal lobar degeneration/ or frontotemporal dementia/ or "pick disease of the brain"/ or lewy body disease/ 96437

32 (dementia or alzheimer*).tw,kw,kf. 0

33 31 or 32 96437

34 Caregivers/ 40101

35 health personnel/ or allied health personnel/ or nursing assistants/ or psychiatric aides/ or exp medical staff/ or exp nurses/ or exp nursing staff/ 64726

36 (health care personnel or nurse* or doctor* or physician* or health care professional* or health care worker*).tw,kw,kf. 0

37 (caregiver* or family or relative* or spouse* or families).tw,kw,kf. 0

38 34 or 35 or 36 or 37 103193

39 India/ 7805

40 (india or indian population*).tw,kw,kf. 0

41 india.in,cp. 42417

42 39 or 40 or 41 50211

43 33 and 38 and 42 51

44 (experience* or perspective* or living).tw,kw,kf. 0

45 43 and 44 0
